# Dietary supplementation with soluble corn fiber improved fecal score, microbiota, and SCFAs in dogs

**DOI:** 10.3389/fvets.2025.1599213

**Published:** 2025-05-20

**Authors:** Donghui Liang, Shuai Zhao, Guoan Yin

**Affiliations:** College of Animal Science and Veterinary Medicine, Heilongjiang Bayi Agricultural University, Daqing, China

**Keywords:** soluble corn fiber, microbial diversity, short-chain fatty acids, dogs, pet food

## Abstract

**Introduction:**

Soluble corn fiber, a safe dietary fiber with prebiotic properties, has been put to several applications in human daily life. However, studies on its use in pet food are scarce. This study was conducted to investigate the effects of SCF on microbial diversity, SCFAs and fecal quality in canines.

**Methods:**

Twenty adult dogs were divided into four groups, including the control group (CON) and three groups fed diet supplemented with 0.1% (SCF1), 0.5% (SCF2), or 1% (SCF3) SCF for 21 days.

**Results:**

Fecal scores of the group fed 1% SCF were the closest to the ideal state. *α*-diversity analysis showed the Chao1 index in the SCF2 and SCF3 groups was significantly higher (*p* < 0.05) than in the CON group, indicating an increase in colony abundance. *β*-diversity analysis showed no significant structural difference among groups (*p* > 0.05). Microbial diversity analysis showed the addition of SCF to the diets increased the relative abundance of *Bacteroidota* and *Blautia* and decreased the relative abundance of *Firmicutes*, *[Ruminococcus]_gnavus_group*, and *Prevotellaceae_Ga6A1_group*; 1% SCF the relative abundance of *Prevotella* and *Blautia* (*p* < 0.05), and the content of acetic acid, valeric acid, and isobutyric acid (*p* < 0.05) and significantly decreased the content of butyric acid (*p* < 0.05).

**Conclusion:**

Dietary supplementation with SCF improves the fecal condition, modulates microbiota composition, enhances the levels of acetic acid, valeric acid, and isobutyric acid, and decreases the level of butyric acid in dogs, with optimal effects observed for 1% supplementation.

## 1 Introduction

With the economic and social development, increasing number of people keep dogs or cats as companion animals. The pet food industry, which accounts for more than two-thirds of the pet economy in China, is witnessing a paradigm shift, with pet owners demanding pet food that not only meets the basic nutrient requirements but also contains health-promoting and disease-preventing ingredients. This change in demand has led to the emergence of functional pet foods, such as prebiotics, probiotics, and synbiotics. As an important prebiotic, dietary fiber can increase the viscosity of the chyme to delay gastric emptying and enhance satiety (1), and prevent constipation by accelerating intestinal peristalsis and increasing fecal volume (2). Additionally, as a fermentation substrate for intestinal flora, dietary fiber promote the proliferation of fiber-utilizing (e.g., *Prevotella* and *Bacteroides*) and beneficial (e.g., *Lactobacillus* and *Bifidobacterium*) bacteria and change the structure of the intestinal flora, the SCFAs produced by their fermentation can reduce intestinal inflammation (3), improve lipid metabolism (4). Soluble corn fiber (SCF), a resistant maltodextrin with prebiotic properties, is a non-viscous, soluble, fermentable dietary fiber prepared from corn starch via thermal and enzymatic treatment under acidic conditions (5). SCF has long been used as a human-edible and safe dietary fiber and has been shown to enhance satiety (6), reduce serum triglyceride and cholesterol levels, improve fecal status, and promote an increase in beneficial intestinal bacteria (7). However, the efficacy of SCF has mostly been investigated in humans, mice, and piglets, and studies on its effects on the microbial diversity in dogs have been scarce. Therefore, this study was aimed at investigating the effects of adding different doses of SCF to canine diets on microbial diversity and short-chain fatty acid production in the intestine of dogs. We hypothesized that (a) SCF would improve fecal conditions and promote the growth of beneficial bacteria in the canine intestine and (b) high doses of SCF would be more beneficial to canine intestinal health than low doses of SCF.

## 2 Materials and methods

### 2.1 Ethics statement

All experiments were approved by and conducted according to the guidelines of the Science and Technology Ethics Committee of Heilongjiang Bayi Agricultural University (DWKJXY2023127).

### 2.2 Experimental design

Twenty adult Pembroke Welsh Corgi, aged 2–6 years, with similar body weights were selected and randomly divided into four groups, with five dogs (3 females and 2 males in each group) in each group. The control group (CON group) was fed only the basal diet, whereas the SCF1, SCF2, and SCF3 groups were fed basal diet supplemented with 0.1, 0.5, and 1% SCF, respectively. The basal ration was provided by Synergistic Biologicals, and its composition and nutrient levels are shown in Supplementary Table S1. SCF was provided by Edymion Biotechnology (Tianjin) Co. The test period was 26 days, of which the pretest period was 5 days, and the main test period was 21 days. The test was carried out at the dog training base of the Jilin Agricultural Science and Technology University. Each dog was kept in a single kennel, fed twice a day, and allowed free access to water.

### 2.3 Sample collection and indicator measurement

#### 2.3.1 Fecal scores

Dogs were observed daily for feces, and scores were recorded, referring the Waltham fecal scoring system in Supplementary Figure S1. Fecal scores on three randomly selected days in weeks 1, 2, and 3 after the formal trial were used for a summary analysis and comparison.

#### 2.3.2 Identification of fecal flora

Fresh feces were collected on day 26 of the experiment and immediately stored in a − 80°C freezer for fecal flora analysis. Fecal DNA was extracted using the MagPure Soil DNA LQ Kit (Magan), the concentration and purity of DNA were measured using a NanoDrop 2000 (Thermo Fisher Scientific, USA) and agarose gel electrophoresis, and the DNA was sequenced by Shanghai Ouyi Biotech Co. (Shanghai Ouyi Biotechnology Co., Ltd.) on an Illumina NovaSeq 6,000 sequencing platform. The reads were first trimmed using the Cutadapt software, and then using DADA2 (8), the qualified paired-end raw reads from the previous step were subjected to quality control analyses, such as quality filtering, noise reduction, splicing and de-chimerization in accordance with QIIME 2 (9) (2020.11), to obtain representative sequences and amplicon sequence variant abundance tables, and all the representative sequences were compared with the Silva (version138) database for annotation. The microbial diversity was estimated using the alpha diversity that include Chao1 (10) and Shannon indices (11). The Unifrac distance matrix performed by QIIME software was used for Principal component analysis (PCA).

#### 2.3.3 Determination of short-chain fatty acid content

On Day 26 of the trial, fresh fecal samples were collected and snap-frozen at −80°C for short-chain fatty acid (SCFA) analysis. The concentrations of acetic acid, propionic acid, butyric acid, valeric acid, isobutyric acid, and isovaleric acid were quantified using ultra-performance liquid chromatography-mass spectrometry (AB Sciex/Waters) following the protocol described by Dobrowolska et al. (12).

### 2.4 Statistical analyses

Data are expressed as mean ± standard error. Raw data were first sorted using Microsoft Excel 2016 and then analyzed using one-way ANOVA with the SPSS 25.0 statistical software. Graphs were prepared using the Graphpad Prism 8.0 software. A value of *p* < 0.05 was considered significant.

## 3 Results

### 3.1 Effect of soluble corn fiber on fecal status in dogs

**Figure 1:**
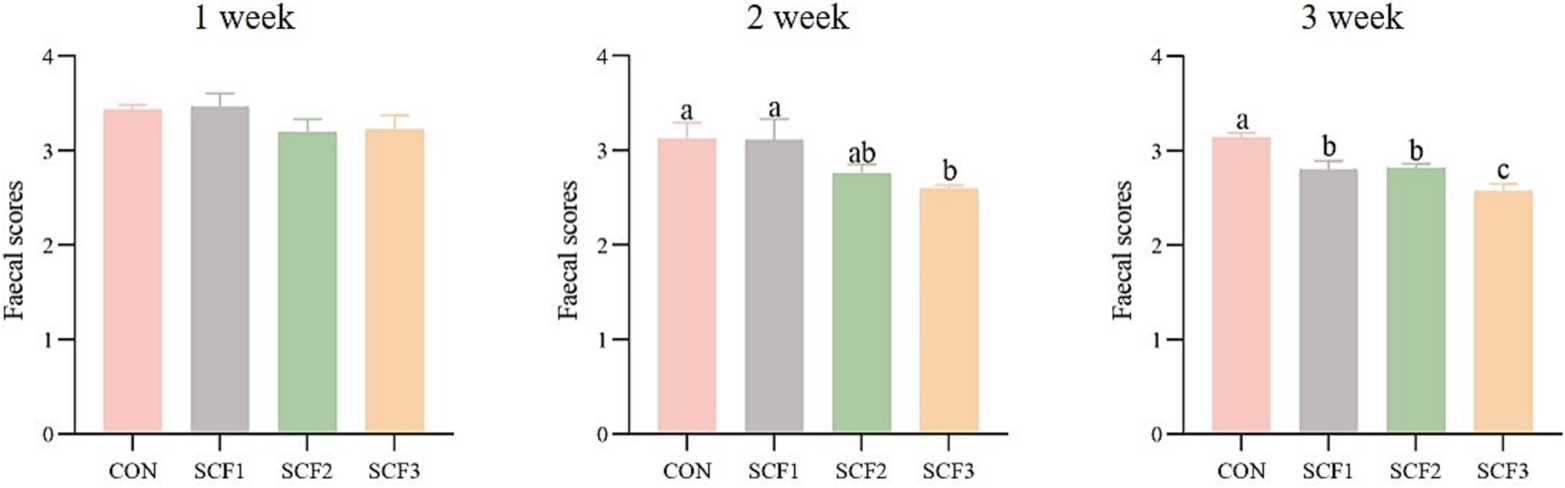
Effect of soluble corn fiber on canine fecal scores.

As shown in Figure 1, the SCF3 group exhibited significantly decreased fecal scores than the control and SCF1 groups (*p* < 0.05) by week 2. By week 3, all treatment groups exhibited significantly lower fecal scores than the control group, and SCF3 groups were significantly lower than SCF1 and SCF2 group (*p* < 0.05), with fecal scores approaching the ideal benchmark of 2.5.

### 3.2 Fecal microbiome analysis

#### 3.2.1 Analysis of fecal microbial diversity

The Chao1 index for SCF2 and SCF3 groups was significantly higher than that for the control group (CON group: 159.38 ± 8.52; SCF1 group: 183.26 ± 13.86; SCF2 group: 194.78 ± 2.09; SCF3: 214.29 ± 12.35, *p* < 0.05), indicating that the addition of higher doses of SCF significantly increased the microbial diversity (Supplementary Table S2). Analysis of the results for *β*-diversity (Supplementary Figure S2) showed no significant differences in microbial diversity among the four groups.

#### 3.2.2 Analysis of the colony structure at the phylum level

**Figure 2:**
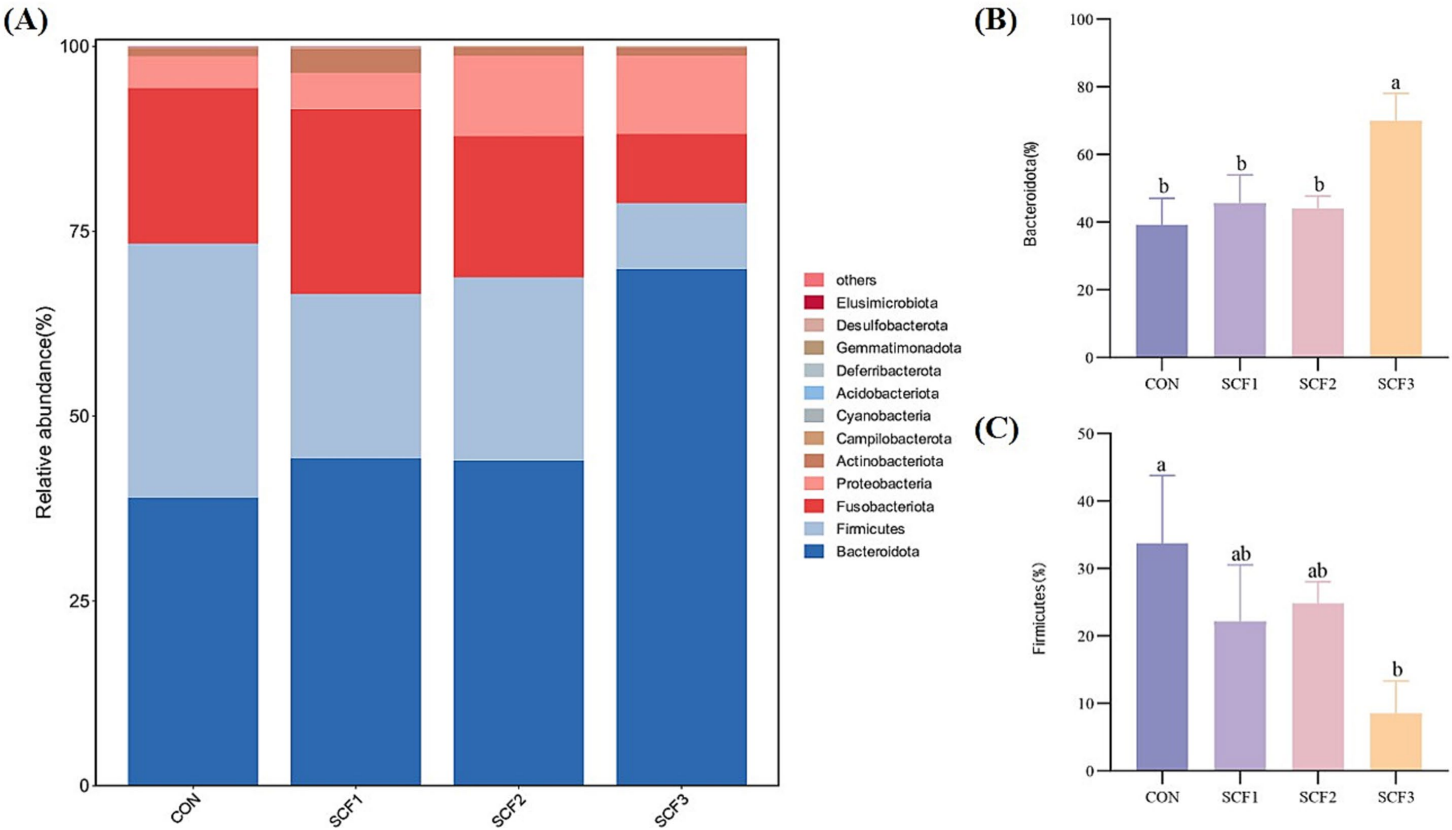
Phylum level analysis of flora. **(A)** Taxonomic bar plot at the phylum level, **(B)** The relative abundance of *Bacteroidota*, **(C)** The relative abundance of *Firmicutes*.

As shown in Figure 2A, the dominant phyla in each group were *Bacteroidota*, *Firmicutes*, and *Fusobacteriota*, with relatively low abundance of *Proteobacteria*, *Actinobacteriota*, and *Campylobacteria*. Figures 2B,C show that the addition of 1% SCF to the diets significantly increased the relative abundance of *Bacteroidota* (*p* < 0.05), and that the relative abundance of *Firmicutes* in the CON group was significantly higher than that in the SCF3 group (*p* < 0.05).

#### 3.2.3 Analysis of the colony structure at the genus-level

**Figure 3:**
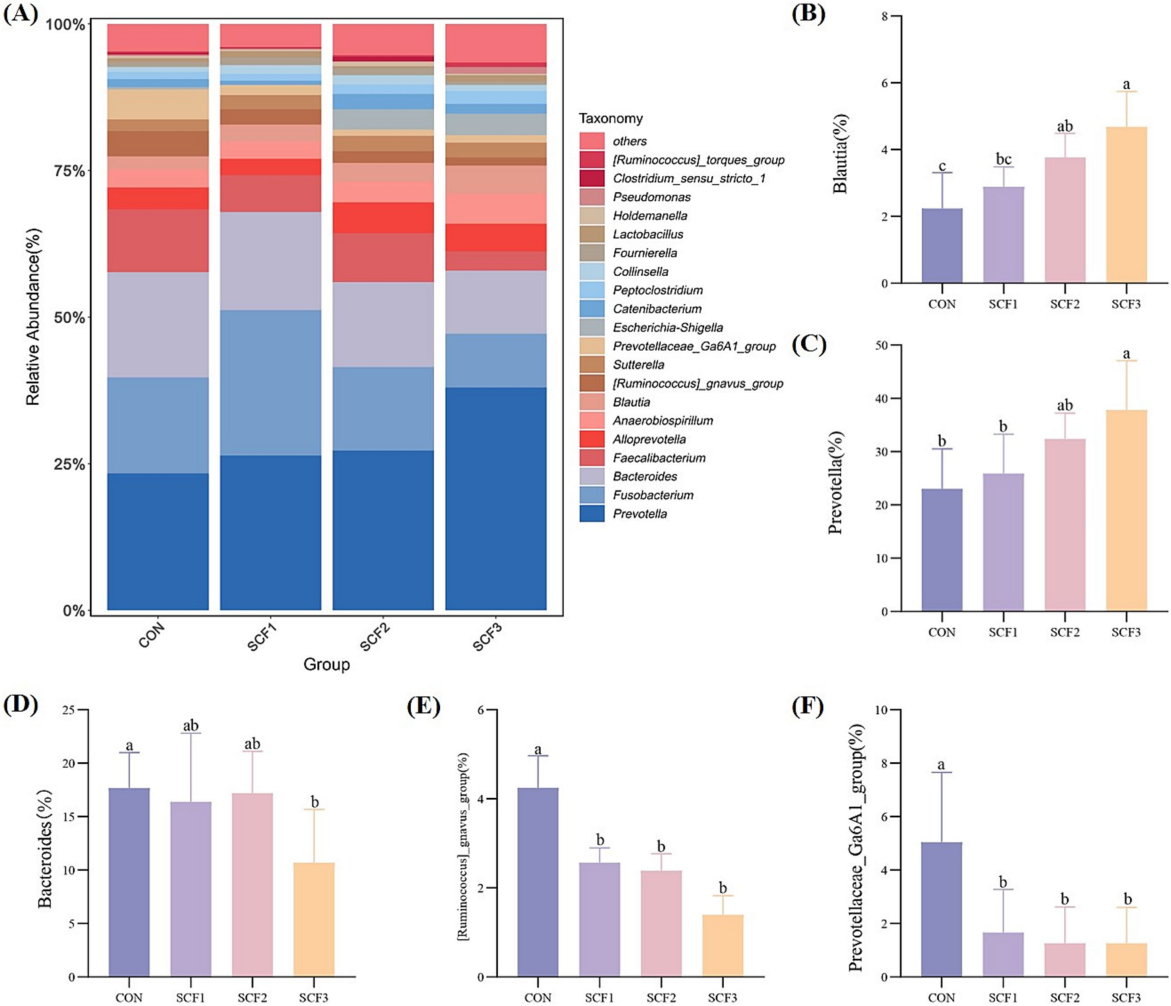
Genus level analysis of flora. **(A)** Taxonomic bar plot at the genus level, **(B)** The relative abundance of *Blautia*, **(C)** The relative abundance of *Prevotella*, **(D)** The relative abundance of *Bacteroides*, **(E)** The relative abundance of *[Ruminococcus]_gnavus_group*, **(F)** The relative abundance of *Prevotellaceae_Ga6A1_group*.

As shown in Figure 3A, the relative abundances of *Prevotella*, *Bacteroides*, *Fusobacterium*, *Faecalibacterium*, and *Alloprevotella*, which are the main microbial diversity genera, were higher in the four groups. The addition of 0.5 and 1% SCF to the diets significantly increased the relative abundance of *Blautia* (*p* < 0.05); the addition of 1% SCF significantly increased the relative abundance of *Prevotella* (*p* < 0.05) and significantly decreased the relative abundance of *Bacteroides* (*p* < 0.05); and in the SCF group the abundance of *[Ruminococcus]_gnavus_group* and *Prevotellaceae_Ga6A1_group* was significantly lower (*p* < 0.05) than in the control group (Figures 3B–F).

### 3.3 Effect of soluble corn fiber on short-chain fatty acids in dog feces

**Figure 4:**
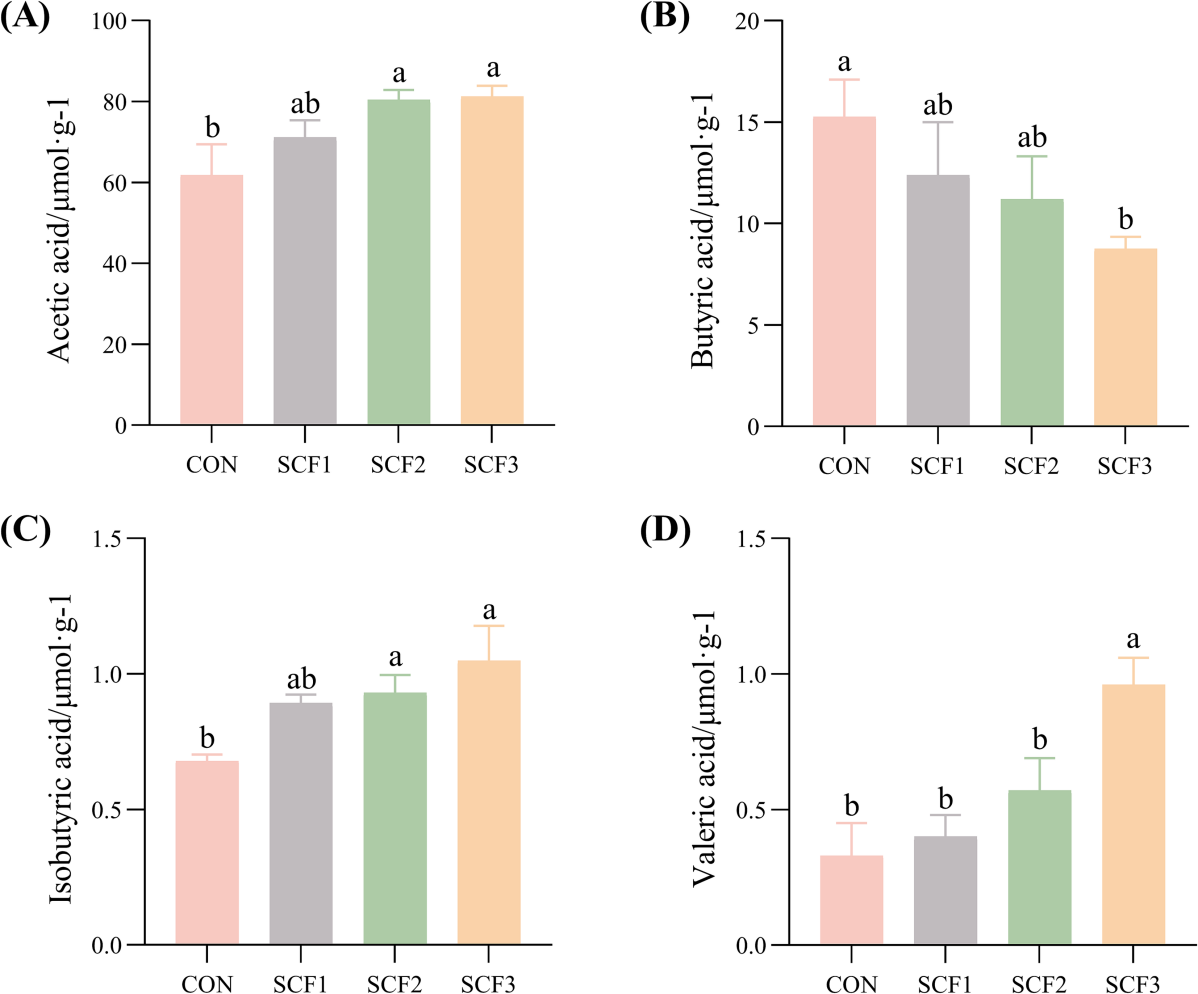
Effect of soluble corn fiber on canine fecal short-chain fatty acid content. **(A)** Acetic acid concentration, **(B)** Butyric acid concentration, **(C)** Isobutyric acid concentration, **(D)** Valeric acid concentration. Different small letters in the same column indicated significant differences between groups (*p* < 0.05).

As shown in Figures 4A–D, the addition of 1% SCF to the diets significantly increased the concentrations of valeric acids in the feces (*p* < 0.05) and significantly decreased the concentration of butyric acid (*p* < 0.05) compared with that in the control group. The concentration of acetic and isobutyric acid in both the SCF2 and SCF3 groups were significantly higher than that in the CON group (*p* < 0.05).

## 4 Discussion

Prebiotics, including oligosaccharides and dietary fibers, are substances that cannot be digested and absorbed by animals; however, their selective utilization by gut microbes improves the intestinal health (13). In present study showed that dietary supplementation with SCF for 2 weeks can improve the fecal condition of dogs, with the SCF3 group exhibiting a fecal score closest to the ideal score of 2.5. This may be attributed to the high viscous and water-holding capacity of SCF, which absorbs water and swells expands in the large intestine, increasing stool bulk and promoting stool formation to improve diarrhea symptoms (14). microbial fermentation of SCF into organic acids help regulate intestinal motility, which could relieve constipation (15). However, a previous study investigating cellulose and prebiotic mixtures studied reported no effect on canine fecal scores (16). This discrepancy may be attributed to the differences in physicochemical properties caused by the structural differences among dietary fibers (such as glycosidic bond and molecular weight). SCF is a dietary fiber that cannot be digested and metabolized by digestive enzymes in the gut, but can only be metabolized by certain species of gut microbiota via anaerobic fermentation, the main products of which are SCFAs. The Chao1 index of alpha diversity was significantly higher in the SCF group, indicating that SCF significantly affected the diversity and abundance of microbes, which is consistent with the findings of Wang et al. (16). At the phylum level, the dominant flora in all groups were mainly *Bacteroidota*, *Firmicutes*, *Fusobacteriota*, and *Proteobacteria*, consistent with the results of a previous study (17, 18). This may be attributed to the fact that the modulation of dietary fiber on bacterial phyla was relatively stable, while more pronounced compositional changes occur at the family, genus or species level. The *Bacteroidota* is involved in the metabolism of a variety of soluble polysaccharides and upregulates the expression of soluble polysaccharide-degrading enzymes (19, 20). The *Firmicutes* have a system for degrading dietary fiber, which tends to transport the fibers into the cell for degradation, which is different from that in *Bacteroidota* (21). In this study, the addition of SCF significantly increased *Bacteroidota* and decreased the abundance of *Firmicutes*, which is in general agreement with the results of Miyazato et al. (22). Some *Blautia* strains can utilize CO, H_2_/CO_2_, and carbohydrates as energy sources (23). Several studies on the utilization of carbohydrates have shown that all strains of the genus *Blautia* can utilize glucose, but different strains differ in their ability to utilize sucrose, fructose, lactose, maltose, and rhamnose, and that they ferment SCFAs, namely acetic, succinic, and lactic acids (24–27). In this study, the addition of a slightly higher dose of SCF to the diet not only significantly increased the relative abundance of *Blautia* but also the level of acetic acid, suggesting that SCF may increase the level of acetic acid in the intestinal tract by promoting the growth of *Blautia* and thus maintain the intestinal health of dogs. Studies showed that acetic acid could modulate adipose tissue inflammation by elevating IL-17 and IL-10 (28), and improve lipid homeostasis via reduction of free fatty acids (e.g., triglyceride and cholesterol) (29). In this study, the addition of 1% SCF increased the relative abundance of *Prevotella* and decreased the relative abundance of *Bacteroides* This finding is consistent with that of Kovatcheva-Datchary et al. (30). *Prevotella* exhibits more superior fiber utilization efficiency than *Bacteroides*, and correlating with enhanced SCFAs production (31). Several enzymes produced by *Prevotella* are involved in polysaccharide degradation and play an important role in the decomposition of polysaccharides and the formation of SCFAs. It has also been demonstrated that *Prevotellaceae_UCG-003* elevate isobutyric acid levels to alleviate diarrhea in piglets (32). In present study, the concentration of isobutyric acid in SCF group increased and the quality of feces were improved. The significantly lower abundance of *Bacteroides* was possibly due to substrate competition with *Prevotella*. The abundance of *[Ruminococcus]_gnavus_group* is positively correlated with chronic inflammatory bowel disease, such as Crohn’s disease (33), In the present study, the abundance of *[Ruminococcus]_gnavus_group* in the dog groups supplemented with SCF was significantly lower than that in the control group, suggesting that consumption of SCF can potentially reduce the risk of inflammatory bowel disease in dogs. The abundance of the *Prevotellaceae_Ga6A1_group* was significantly lower in the SCF group than in the control group, which is consistent with the findings of Thunyaporn et al. (34). In present study, isobutyric and valeric acids concentrations were elevated and butyrate acids concentration was reduced in the 1% SCF group than in the control group. Butyrate acids is the primary energy substrate for colonic epithelial cells. Butyric acid produced by the fermentation of resistant starch can promote intestinal peristalsis (35). The decrease in butyric acid concentration in present study might be related to the accelerated utilization of intestinal epithelial cells. Several studies have shown that the addition of dietary fibers with prebiotic potential to the diet promotes the production of isobutyric and valeric acids (36–38), which is consistent with the findings of the present study. As energy substrates for colonic epithelial cells, isobutyric and valeric acids regulate intestinal motility and fecal excretion (39). The addition of valeric acid derivatives also showed positively effects on the mucosa (40). In this study, the optimal addition amount of SCF could not be determined because only three gradients of SCF addition were set, which failed to fully cover its dose-effect relationship. In addition, the experimental results showed that the abundance of *Proteobacteria* increased significantly after the addition of SCF, and the specific mechanism of this phenomenon is not clear, which may be related to the dose-dependent flora response or individual differences. In order to further validate this result and explore the potential mechanism, it is recommended to expand the sample size and set a wider dose gradient in subsequent studies to comprehensively evaluate the effects of SCF on intestinal flora and host metabolism.

## 5 Conclusion

Adding SCF to the diet for 3 weeks improved the fecal condition, modulated microbiota composition and enhanced SCFA production in dogs, with optimal effects observed at 1% inclusion level. These findings provide a supporting basis for the application of SCF in pet food.

## Data Availability

The data presented in this study are deposited in the NCBI repository, with the accession number PRJNA1263151. Further inquiries can be directed to the corresponding author.
