# Supplementary material for: Dietary supplementation with soluble corn fiber improved fecal score, microbiota, and SCFAs in dogs
Source: Front Vet Sci. 2025 May 20;12:1599213. doi: 10.3389/fvets.2025.1599213 (PMC12131326; doi:10.3389/fvets.2025.1599213)
Supplement: Supplementary file 1 [file Table_1.DOCX]

Supplementary Material

**Table S1** Nutrient levels of basal rations (dry matter basis, %)

| Nutrient composition | Level |
| --- | --- |
| Crude protein | 20.86 |
| Ether extract | 8.4 |
| Crude fiber | 3.3 |
| Crude ash | 6.2 |
| Calcium | 0.98 |
| Total phosphorus | 0.76 |

**Table S2** Alpha diversity indices of gut microbiota

| Group | ACE | Chao1 | Shannon | Simpson |
| --- | --- | --- | --- | --- |
| CON | 139.72±13.55 | 159.38±8.52^b^ | 5.02±0.12 | 0.95±0.01 |
| SCF1 | 142.32±32.33 | 183.26±13.86^ab^ | 5.21±0.21 | 0.92±0.02 |
| SCF2 | 144.46±5.03 | 194.78±2.09^a^ | 5.47±0.07 | 0.93±0.01 |
| SCF3 | 191.18±36.10 | 214.29±12.35^a^ | 5.48±0.09 | 0.91±0.01 |

Note: Different small letters in the same column indicated significant differences between groups (*p* < 0.05).


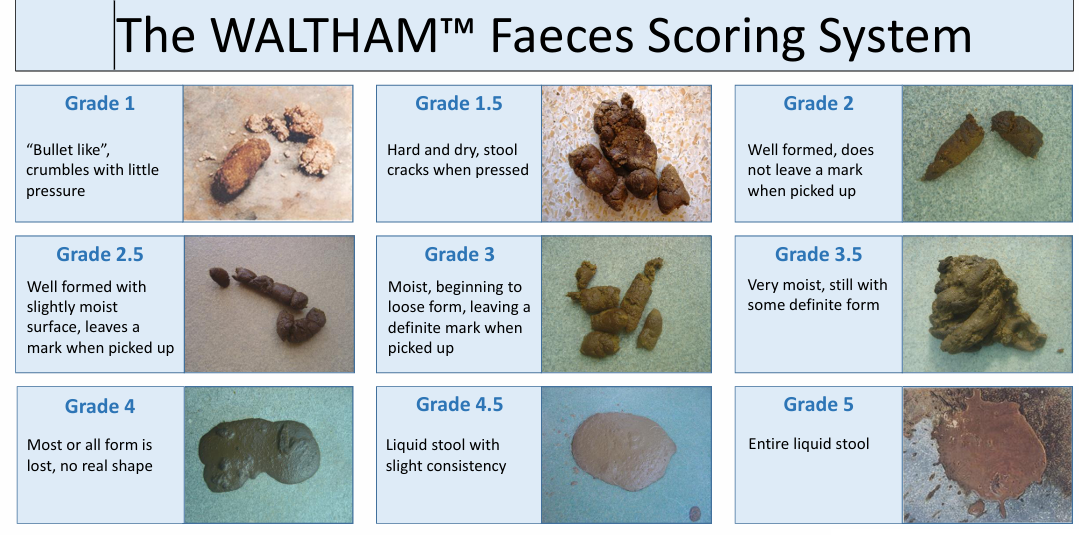


**Supplementary Figure 1.** The WALTHAM™ Faeces Scoring System


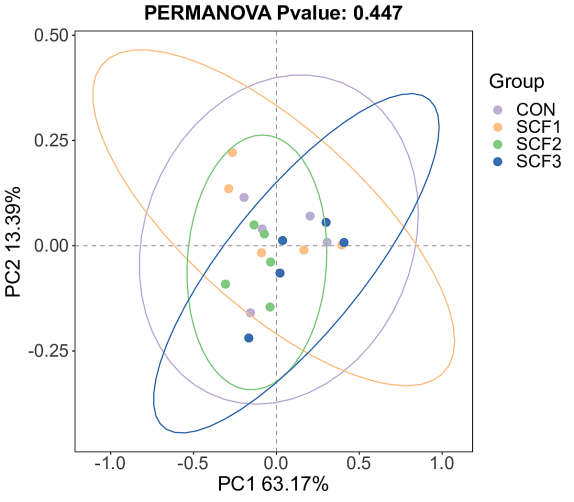


**Supplementary Figure 2.** PCA Analysis
